# Prostatic lithiasis complicating granulomatous prostatis of tuberculous origin: About a case report

**DOI:** 10.1016/j.eucr.2021.101875

**Published:** 2021-10-01

**Authors:** Ramzi Mejri, Kays Chaker, Mokhtar Bibi, Sami Ben Rhouma, Yassine Nouira

**Affiliations:** a Departement of Urology, Hospital Mongi Slim La Marsa, Tunisia; b Departement of Urology, La Rabta Hospital, Tunisia

**Keywords:** Prostate, Lithiasis, Tuberculosis, Lithotripsy

## Abstract

Urogenital tuberculosis remains a frequent disease in our country. It is the most common extra-pulmonary location. Prostatic involvement is extremely rare.

We report the observation of a prostatic lithiasis complicating a granulomatous prostatitis of tuberculous origin, revealed essentially by obstructive and storage lower urinary tract symptoms. The diagnosis was suspected on imaging and clinical findings and confirmed by histology. Treatment consisted of endoscopic resection of the prostate associated with endoscopic ballistic lithotripsy.

Prostatic lithiasis is a rare condition with a poorly elucidated ethiopathogeny. The origin of tuberculosis should be evoked in front of this affection.

## 1 Introduction

Urogenital tuberculosis remains a frequent disease in our country. It is the most frequent extra pulmonary localization. Prostatic involvement is extremely rare as shown by the scarcity of observations published in the literature.

We propose to review this pathology in the light of the observation of a prostatic lithiasis complicating a prostatic granulomatosis of tubercular origin revealed by lower urinary tract symptoms.

## 2 Case report

A 48-year-old men, with a medical history of treated cerebral tuberculosis, presented to our consultation unit with a symptomatology that had been evolving for 3 months, consisting of storage and obstructive lower urinary tract symptoms such as urgency, frequency, nocturia three times/night and poor stream without any notion of hematuria.

**Fig. 1:**
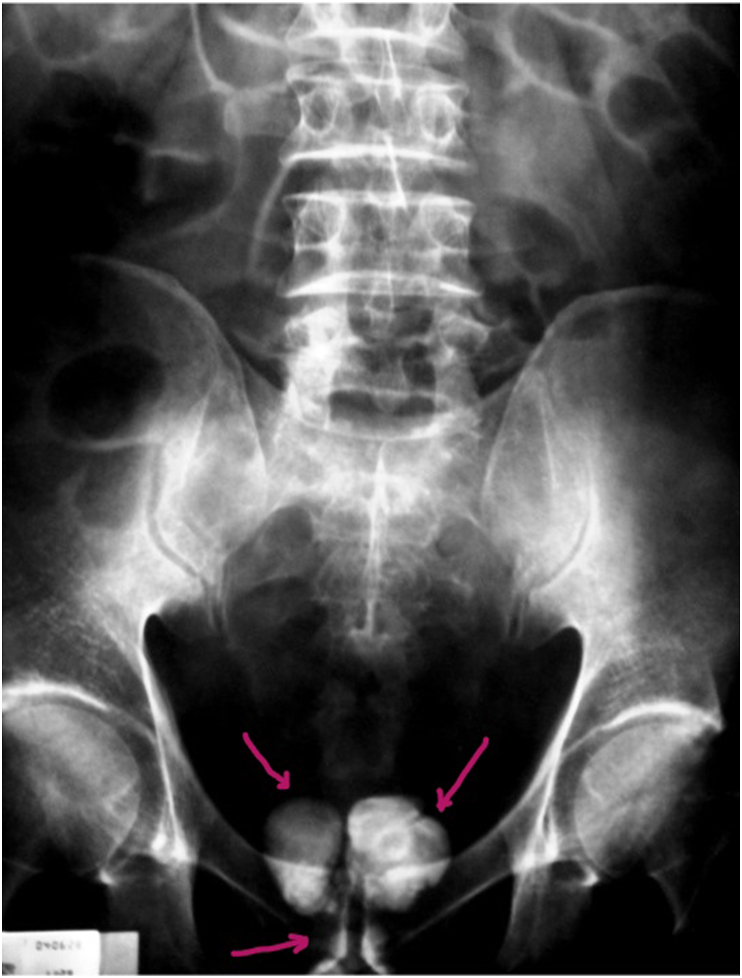
KUB radiography: Bilobed opacity of calcic tone projecting on the prostatic cavity, two other opacities projecting on the path of the urethra.

**Fig. 2:**
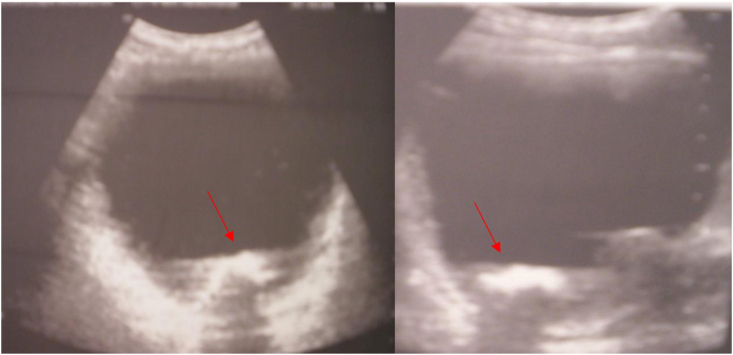
Transabdominal ultrasound of the bladder and prostate: Several hyperechoic formations with posterior shadow cone suggesting prostatic lithiasis.

**Fig. 3:**
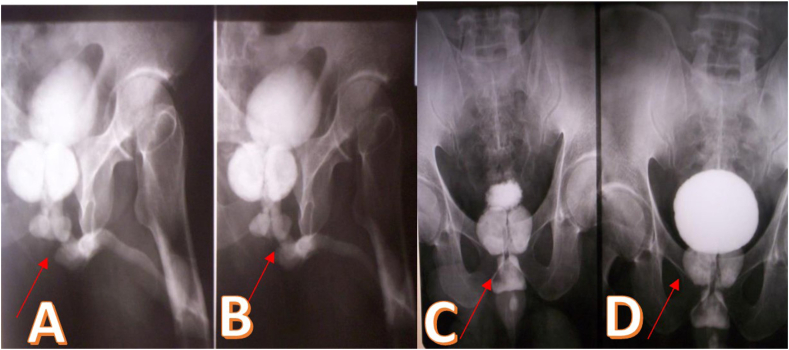
a and b. Retrograde urethrogram: pre-bulbar urethral stricture. c and d. Retrograde urethrogram: Presence of large prostatic lithiasis.

Clinical examination found a patient in good general condition with an indurated nodule in the tail of the right epididymis. The rectal examination reveals a multinodular prostatic hypertrophy, of hard consistency and irregular contours measuring 50 g. The cytobacteriological analysis of the urine was normal as well as his renal function (the research of Koch bacillus in the urine three days in a row was negative). The PSA value was normal. KUB radiography showed the presence of a bilobed calcification projecting on the prostatic cavity associated with two small centimetric lithiasis projecting on the path of the prostatic urethra (Fig. 1). Transabdominal ultrasound of the bladder and prostate revealed several hyperechoic formations with a posterior shadow cone suggestive of prostatic calculi (Fig. 2). Intravenous urography was performed showing several prostatic calcifications and absence of radiological signs suggestive of urogenital tuberculosis. In view of an etiological assessment, we completed our radiological investigations with retrograde urethrogram which showed the presence of a pre-bulbar urethral stricture with enormous bilobed prostatic lithiasis (Fig. 3).

The patient was operated on after a correct pre anesthetic workup. He had a urethrocystoscopy which showed a pre-bulbar urethral stricture not very tight and unifocal, a prostate swollen by multiple stones (highlighted after endoscopic resection of the prostatic lobes). The treatment consisted of an internal urethrotomy for the urethral stricture, an endoscopic resection of the prostate associated with endoscopic ballistic lithotripsy. The patient had almost complete fragmentation of the large lithiasis fragments in the prostatic compartment. The stones were left in intimate contact with the prostatic capsule. The macroscopic aspect of the stones is in favor of brushite stones. The anatomopathological study of the resection shavings was in favor of prostatic tuberculosis (epithelio-giganto-cellular granuloma without caseous necrosis).

The patient was put on antituberculosis treatment for 6 months combining streptomycin, rifampicin, isoniazid and pyrazinamide.

The evolution was marked by the recurrence of irritative lower urinary tract symptoms. Radiological explorations (Retrograde urethrogram) concluded to a sclerosis of the bladder neck and the patient underwent an endoscopic resection of the bladder neck. After the endoscopic intervention the patient became asymptomatic. One year later, the patient is satisfied with his urination with a correct flow meter curve.

## 3 Discussion

Urogenital tuberculosis is still common in our climate and its incidence is increasing worldwide. It ranks fifth after pulmonary, lymph node, osteoarticular and digestive localization. The prostatic localization is rare. This rarity is underlined by the majority of authors.^1^ The possible routes of contamination of the prostate by Koch's bacillus are the descending route from the urinary tract following the course of urine in the excretory cavities, the intra-canalicular route by contiguity from an adjacent tuberculous focus in the genital tract and the hematogenous route.^2^ The latter seems to be the most likely route of dissemination for most authors. However, the hypothesis that genital tuberculosis is a sexually transmitted disease is possible, especially since live Koch bacilli have been demonstrated in semen in cases of prostatic tuberculosis. In rare cases, authors have reported granulomatous prostatitis induced by intravesical BCG therapy for superficial bladder carcinoma.^3^ The histological lesions of prostatic tuberculosis have no particular character (typical tubercular follicles grouped in granulation or a nodule). The elementary lesion is an epithelio-giganto-cellular granuloma with caseous necrosis. The macroscopic aspect depends on two opposite processes, a mechanism of destruction and caseation creating the cavities and a defense process by fibrosis limiting the extension of the lesions. Lithiasis can develop within the prostatic tissue (acini, ducts) and should be distinguished from stones embedded in the prostatic urethra. These primary endogenous prostatic stones are formed from the amylaceous bodies often present in the acini. The stasis of prostatic secretion resulting from obstruction, inflammation and infection of the prostatic ducts promote the formation of stones. Stones can form inside the prostatic tubercular caverns, as was the case for our patient. Clinically, lower urinary tract symptoms are often in the foreground. A hematuria, a urethrorrhagia, a hemospermia can reveal prostatic lithiasis. The digital rectal examination may reveal an enlarged prostate with an elastic, firm or even stony consistency. The perception of small hard nodules separated from healthy tissues on the digital rectal examination is in favor of the diagnosis of lithiasis. Nevertheless, the digital rectal examination data are not specific and can lead to confusion with an adenoma or prostate cancer.^4^ The diagnosis of prostatic tuberculosis is based on the search for Koch's bacillus in the urine or in the seminal fluid. Preoperative urine tests (AFB tests tuberculosis) are rarely positive in the case of prostate tuberculosis with a positivity rate of about 20%.^3^ Prostate biopsy is the diagnostic tool of choice. Treatment is primarily medical, based on the use of anti-tuberculosis drugs for six months according to the following regimen: two months of quadruple therapy (streptomycin, rifampicin, isoniazid and pyrazinamide) and four months combining isoniazid and rifampicin. The treatment of giant prostate stones is performed by open surgery (radical prostatectomy, retro pubic prostatolithotomy or cystolithotomy with incision of the bladder neck).

The transurethral endoscopic approach is simple to perform. It is less invasive and reproducible in case of recurrence. It is more suitable for small and multiple prostatic calculi.^5^

## 4 Conclusion

Prostatic lithiasis is a rare pathology of poorly elucidated etiology. The origin of tuberculosis should be evoked in front of this affection in spite of the rarity of the localization of this disease. The treatment consists of endoscopic resection of the prostate generally associated with ballistic lithotripsy.

## Declaration of competing interest

The authors declare that there are no conflicts of interest regarding the publication of this article.
